# Marginal Misfit Assessment in Common Restorative Materials for Cement‐Retained Fixed Implant Prostheses: An In Vitro Comparative Study

**DOI:** 10.1002/cre2.70318

**Published:** 2026-02-25

**Authors:** Pedro Diaz, Barbara Miegimolle, Antonio Martin, Seyed Ali Mosaddad, Celia Tobar, Maria J. Suarez

**Affiliations:** ^1^ Department of Conservative Dentistry and Bucofacial Prostheses, Faculty of Odontology Complutense University of Madrid Madrid Spain; ^2^ Department of Clinic Grade Dentistry, Faculty of Odontology European University of Madrid Madrid Spain; ^3^ Department of Research Analytics, Saveetha Dental College and Hospitals, Saveetha Institute of Medical and Technical Sciences Saveetha University Chennai India; ^4^ Department of Prosthodontics, School of Dentistry Shiraz University of Medical Sciences Shiraz Iran

**Keywords:** abutments, CAD‐CAM technologies, crowns, dental implants, marginal misfit

## Abstract

**Objectives:**

To assess the vertical discrepancy at the abutment–crown interface (ACI) for cement‐retained crowns fabricated from three commonly used restorative materials across implants featuring external and internal connections.

**Materials and Methods:**

A total of 30 implants with external (EC) and internal (IC) connections were included in this study. Each implant received a prefabricated titanium abutment, which was randomly attached using the torque recommended by the respective manufacturers. Three types of restorative materials—metal‐ceramic (MC), monolithic zirconia (MZ), and veneered zirconia (VZ)—were used to fabricate the crowns for each connection type and cemented to their respective abutments. Following 6000 cycles of thermal cycling between 5°C and 55°C, the vertical marginal misfit was evaluated using scanning electron microscopy. Data were statistically analyzed using repeated‐measures ANOVA, followed by Bonferroni post‐hoc test and paired *t*‐test.

**Results:**

The lowest marginal misfit was recorded for VZ restorations. Although the IC group showed lower mean misfit values compared to the EC group, this difference was not statistically significant (*p* = 0.980). Significant differences were found in the IC group between MZ and VZ (*p* = 0.016) and between MC and VZ (*p* = 0.035). Mean misfit values (µm) for each material were as follows: VZ = 18.7 (SD = 4.5), MZ = 42.2 (SD = 8.1), and MC = 39.9 (SD = 7.4).

**Conclusions:**

Cement‐retained veneered zirconia restorations demonstrated less vertical ACI misfit compared to metal‐ceramic and monolithic zirconia restorations. The connection configuration did not influence the marginal misfit. All measured misfit values were below 50 µm.

## 1 Introduction

Dental implants offer both functional and aesthetic rehabilitation of edentulous spaces; however, their long‐term success depends not only on osseointegration but also on prosthetic factors (Buser et al. 2017; Mosaddad et al. 2024). Since Brånemark's introduction of osseointegration, ongoing advancements have improved implant systems and prosthetic workflows. Nevertheless, achieving an accurate marginal adaptation at the abutment–crown interface (ACI) remains a clinical challenge, as discrepancies at this interface can lead to mechanical failures such as screw loosening, abutment fracture, retention loss, and biological complications including peri‐implantitis (Abdelrehim et al. 2024; Astolfi et al. 2022; French et al. 2021; Jemt 2017; Schwarz and Ramanauskaite 2022). Marginal misfit, defined as the vertical or horizontal gap between the crown margin and the abutment finish line, is influenced by several interrelated factors, including the design of both the restoration and abutment, cementation technique, veneering protocol, and material properties such as low‐temperature degradation of zirconia (Del Piñal et al. 2021; Gonzalo et al. 2009b; Musleh et al. 2020; Yildirim 2020). Sultan et al. have recommended a minimum cement gap of 60 µm to ensure adequate prosthesis adaptation (Sultan et al. 2021). However, the absence of standardized in vitro and in vivo assessment protocols continues to limit the establishment of evidence‐based clinical thresholds, leading to heterogeneous findings (Katsoulis et al. 2017; Mai et al. 2020; Pan et al. 2021).

The implant–abutment connection plays a key role in maintaining mechanical stability and preserving peri‐implant bone. Among implant–abutment configurations, internal connections (IC)—particularly conical or Morse taper designs—have become increasingly prevalent due to their deeper engagement, frictional locking, and superior resistance to micromovement and rotational forces (Ceruso 2017; D'ercole et al. 2022; Gehrke et al. 2022; Vinhas et al. 2020). These features contribute to improved stress distribution and enhanced long‐term mechanical and biological stability (Caricasulo et al. 2018; Schmitt et al. 2014). By contrast, external connections (EC), the earliest designs, rely solely on screw retention, which under functional loading may cause the abutment to act as a pivot point—concentrating stress and increasing the risk of micro‐movements and screw loosening, especially under non‐axial forces (Caricasulo et al. 2018; Pjetursson et al. 2018). Despite their declining popularity in contemporary practice, EC systems remain relevant for comparative analysis as they are still used in certain clinical scenarios, educational institutions, and legacy implant systems. Including EC in experimental studies enables benchmarking and broader interpretation of connection‐type influence under standardized protocols.

Equally critical to marginal fit is the selection of restorative material. Metal‐ceramic (MC) crowns remain a widely used option due to their strength and reliability, whereas zirconia‐based restorations—especially monolithic zirconia (MZ) and veneered zirconia (VZ)—have gained traction due to their favorable esthetic and mechanical properties (Lemos et al. 2019; Sailer et al. 2015). MZ restorations offer high fracture resistance and longevity, while VZ restorations are often preferred in esthetically demanding cases due to their superior translucency and layered anatomy (Ansari et al. 2024; Dini et al. 2021; Pjetursson et al. 2018). These materials differ in their thermal behavior, processing sensitivity, and structural composition, which can influence marginal accuracy. For instance, MC crowns may exhibit increased misfit due to multiple firing cycles, porcelain shrinkage, and mismatches in the coefficient of thermal expansion (CTE) between metal and ceramic components (Abduo et al. 2023; Alsarani et al. 2023; Elter et al. 2024; Torabi et al. 2015). MZ restorations, though more dimensionally stable during sintering, are subject to significant volumetric shrinkage and may deform during milling or sintering if not properly compensated. VZ crowns, on the other hand, combine a zirconia core with feldspathic veneering layers; although veneering can introduce distortion, controlled layering and the compressive forces generated during cooling may improve marginal adaptation. Moreover, the high fracture toughness of zirconia copings may resist deformation during firing, potentially yielding better marginal precision (Ansari et al. 2024; Dini et al. 2021; Pjetursson et al. 2018).

Advances in CAD/CAM technologies have improved the manufacturing precision of these materials, supporting their clinical application (Sadeqi et al. 2021). Nevertheless, comparative evidence regarding their marginal behavior when combined with different implant connection types remains limited. Most prior studies have focused on either material or connection type in isolation, often with inconsistent methodologies (Katsoulis et al. 2017; Mai et al. 2020; Pan et al. 2021). The indirect influence of implant–abutment connection geometry on crown adaptation, although often overlooked, may be clinically relevant. Even when using prefabricated titanium abutments, the connection configuration determines the vertical stop, rotational stability, and seating precision of the abutment, which may, in turn, affect how the crown seats during cementation. For instance, ICs may minimize torque‐induced vertical micro‐movements during abutment placement and provide a more reproducible seating platform compared to ECs (Gupta et al. 2015).

Given these considerations, the present in vitro study aimed to evaluate the vertical marginal misfit at the abutment–crown interface for cement‐retained crowns fabricated from three commonly used restorative materials—metal‐ceramic, monolithic zirconia, and veneered zirconia—on implants with internal and external connection configurations. The null hypothesis was that neither the restorative material nor the implant–abutment connection type would significantly influence the vertical marginal misfit at the abutment–crown interface.

## 2 Materials and Methods

### 2.1 Fabrication of Specimens

Sixty specimens were fabricated from solid, machined acrylic (PERSPEX XT; 3 A Composites GmbH, Singen, Germany). The sample size was determined based on prior in vitro studies (Diaz et al. 2025; Gonzalo et al. 2020). Using a power analysis with an *α* = 0.05, power = 0.8, and a moderate effect size = 0.25, a minimum of 10 samples per subgroup was calculated to detect significant differences between the study variables, namely, type of material (MC, MZ, VZ), implant connection (EC, IC), and measurement surface (B, L). The specimens were designed in AutoCAD version 14R (Autodesk, San Rafael, CA) and served as retention bases for implant placement. Each specimen had dimensions of 15 mm in width, 15 mm in length, and 10 mm in height, with a central perforation of 3.4 mm for implant placement. To ensure uniform implant placement, a central point was marked during the machining process.

### 2.2 Sample Preparation and Grouping

**Figure 1:**
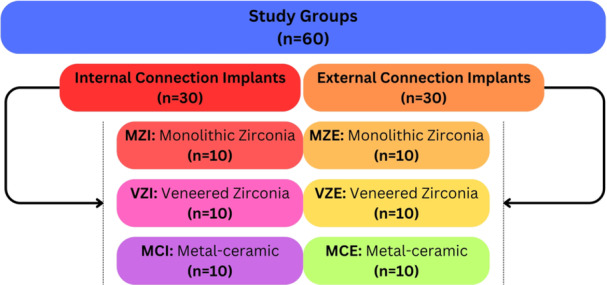
Schematic overview of the study design, illustrating the six experimental groups based on implant connection type (internal or external) and restorative material (monolithic zirconia, veneered zirconia, or metal‐ceramic). Each subgroup consisted of 10 specimens.

The specimens were divided into three main groups (*n* = 20 per group) according to the restorative material used: MC, MZ, and VZ. Each material group was subsequently subdivided into two subgroups (*n* = 10) based on the implant connection type: IC and EC. The resulting subgroups were MCI, MZI, VZI (IC), and MCE, MZE, VZE (EC), as illustrated in Figure 1. Detailed specifications of the implants, abutments, and restorative materials used in each subgroup are provided in Table 1.

**Table 1:** Details of the materials used in the study.

Implant connection type	Implant model and brand	Implant dimensions (diameter, prosthetic platform, and length)	Abutment type and brand	Abutment dimension	Restoration type	Core material	Chemical composition	Veneering ceramic
Internal hexagon	Tapered Screw‐Vent (Zimmer Biomet, USA)	Ø 3.7 mm, Ø 3.5, 8 mm	Hex‐Lock Contour Abutments (Zimmer Biomet, USA) Titanium Ti‐6A1‐4V	Emergence profile 4.5 mm; cuff height 3 mm; shoulder width 2 mm	Monolithic zirconia	Lava Plus (3M ESPE, Seefeld, Germany)	3Y‐TZP high translucency zirconia	N/A
					Metal‐ceramic	Coron (Straumann, Basel, Switzerland)	60.5% Co, 28% Cr, 8.5% W, 1.65% Si, and trace amounts (< 1%) of Mn, N, Nb, and Fe.	Vita VM13 (VITA Zahnfabrik, Bad Säckingen, Germany) feldspathic veneering ceramic
					Ceramic‐veneered zirconia	Lava Zirconia system (3M ESPE)	3Y‐TZP zirconia	Lava Ceram (3M ESPE) feldspathic veneering ceramic
External hexagon	TSH S3 (Phibo, Barcelona, Spain)	Ø 3.6 mm, Ø 4 mm, 8.5 mm	TSH Hexed Abutment Post (Phibo, Barcelona, Spain) Titanium Ti‐6A1‐4V	Emergence profile 5 mm; cuff height 3 mm; shoulder width 2 mm				

Abbreviations: 3Y‐TZP, 3 mol% yttria‐stabilized tetragonal zirconia polycrystal; Co, cobalt; Cr, chromium; Fe, iron; Mn, manganese; N, nitrogen; N/A, not applicable; Nb, niobium; Si, silicon; W, tungsten.

### 2.3 Implant and Abutment Placement

**Figure 2:**
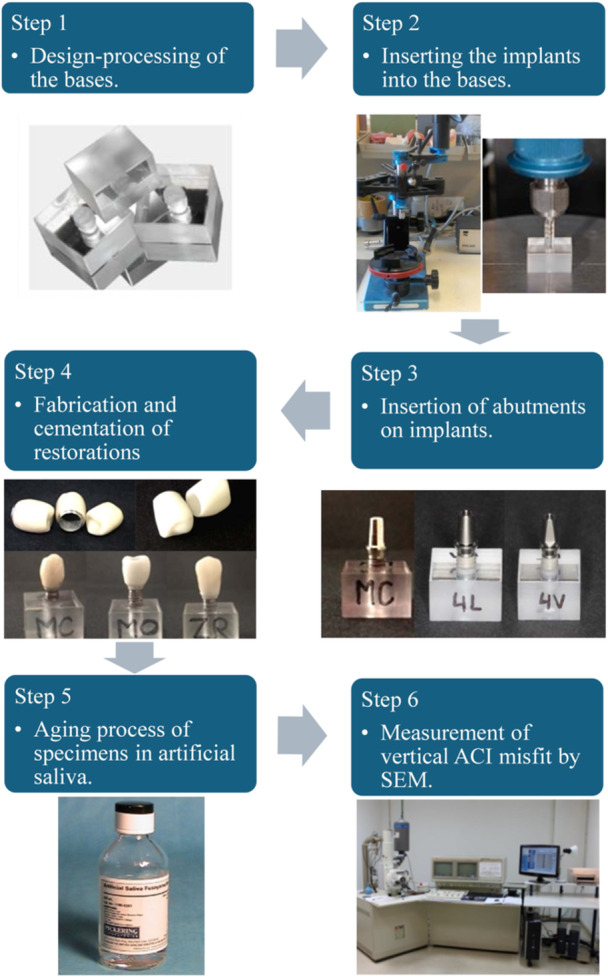
Workflow diagram outlining the experimental methodology, including specimen preparation, implant placement, crown fabrication, cementation, aging procedures, and marginal fit analysis.

Implant beds were prepared using a parallel drilling machine (PFG 100; Cendres & Metaux SA, Biel‐Bienne, Switzerland) to replicate the surgical preparation, ensuring consistent implant placement and angulation. The drilling process followed the specific drilling protocols recommended by the manufacturers of the implant systems. Implants were then inserted at the level of the bone crest using a torque‐controlled wrench, following the manufacturer's guidelines. Abutments were manually tightened by a single operator using calibrated torque wrenches (30 Ncm for Zimmer abutments and 35 Ncm for Phibo abutments). Figure 2 illustrates the complete methodological process used throughout the study.

### 2.4 Restoration Fabrication

The crowns were designed using a computer‐aided design and computer‐aided manufacturing (CAD‐CAM) workflow. In the MC group, implant abutments were scanned with a desktop scanner (CARES CS2; Straumann, Basel, Switzerland). The frameworks, with a thickness of 0.5 mm and a cement space of 50 µm, were digitally designed and milled from cobalt‐chromium blocks (Coron; Straumann) using a 5‐axis milling machine (CORiTEC 350i x pro; imes‐icore, Eiterfeld, Germany). The surface of the framework was prepared using tungsten carbide burs to optimize the bonding surface for the veneering material. The veneering was performed using a feldspathic ceramic (VITA VM13; VITA Zahnfabrik, Bad Säckingen, Germany), in accordance with the manufacturer's guidelines, following a firing sequence at 920°C (opaquer), 880°C and 860°C (dentin), and 800°C (glaze).

VZ crowns were fabricated using the Lava Zirconia system (3M ESPE; Seefeld, Germany). The process began by scanning the abutments with an optical scanner (Lava Scan; 3M ESPE). The frameworks were designed (Lava CAD; 3M ESPE), with a uniform 0.5 mm thickness, a 0.35 mm reinforced edge, and a 50 µm cement space extending 2.3 mm coronally. Designs were milled from pre‐sintered zirconia blocks (Lava Form; 3M ESPE), with a 20% magnification factor to account for sintering shrinkage. After milling, the frameworks were separated from the zirconia block, polished at low speed to eliminate sharp edges or grooves, and sintered at 1500°C for 11 h, including a 3.5‐h drying phase (Lava Therm; 3M ESPE). Following sintering, the frameworks were polished using fine‐grain diamond burs, ultrasonically cleaned, and then veneered (Lava Ceram; 3M ESPE). A liner layer (0.1–0.2 mm thick) was applied and fired at 820°C (Programat P500; Ivoclar Vivadent, Schaan, Liechtenstein), followed by dentin and incisal layers at 810°C and 800°C, respectively, and a final glaze layer at 790°C. Veneering thicknesses were 0.5 mm (axial) and 2 mm (occlusal). MZ crowns were fabricated using the Lava Plus system (3M ESPE), following a similar workflow to the veneered restorations but omitting the veneering phase. The same laboratory and technician fabricated all restorations to ensure consistency and uniform quality.

### 2.5 Cementation of Restorations

All implant components and prosthetic restorations used in this study were newly fabricated specifically for this investigation. No specimens or abutments were reused or recycled. Each crown was cemented to a prefabricated titanium abutment only once, and no re‐cementation or reprocessing was performed at any point. Within each group, crowns were randomly assigned to prefabricated titanium abutments using a computer‐generated random number sequence to avoid selection bias and ensure balanced distribution.

Prior to cementation, all abutments were cleaned in an ultrasonic bath (Isolab Laborgeräte GmbH, Germany) containing 96% ethanol for 5 min to eliminate surface contaminants. Following cleaning, the abutment surfaces were abraded using airborne particles of 50 µm aluminum oxide at a pressure of 2 bar for 10 s, held at a 10 mm distance. This protocol was selected to enhance surface energy and micromechanical retention of the luting agent (Zahoui et al. 2020). For the inner surfaces of the zirconia crowns (both monolithic and veneered), a tribochemical silica coating (CoJet System; 3M ESPE, Seefeld, Germany) was applied under the same conditions (50 µm, 2 bar, 10 s) followed by silane application (ESPE‐Sil; 3M ESPE) for 60 s and gentle air drying. The metal‐ceramic crowns underwent sandblasting with 50 µm alumina and no silane was applied, following conventional clinical protocols for metal‐based restorations.

All crowns were cemented using a dual‐cure self‐adhesive resin cement (RelyX Unicem; 3M ESPE), mixed in accordance with the manufacturer's recommendations. The cement capsules were activated (3M ESPE activator), mixed for 10 s using a Rotomix device (3M ESPE), and loaded into an Aplicap applicator (3M ESPE). Cement was applied to the internal axial walls of each crown, which was then seated on the abutment under a constant pressure of 10 N using a calibrated dynamometric key (USAG 820/70; SWK Utensilerie, Milan, Italy) for 10 min. Excess cement was meticulously removed using an explorer under 5× magnification.

### 2.6 Aging Procedure

Specimens were subsequently stored in a cylindrical polyethylene container containing 30 mL of artificial saliva (Fusayama‐Meyer; LCTech, Obertauftkirchen, Germany) (Rodríguez et al. 2021; Schiff et al. 2002) and subjected to thermal cycling in a climatic chamber (CCK0/81; Dycometal, Barcelona, Spain), alternating between 5°C and 55°C for a total of 6000 cycles, considered equivalent to approximately 6–8 months of clinical function (Antanasova et al. 2018; Kim et al. 2023). This process was controlled using iTools software (Eurotherm; Schneider Electric, Madrid, Spain).

### 2.7 Marginal Fit Analysis

All specimens were sputter‐coated with gold to ensure adequate surface conductivity and imaged using scanning electron microscopy (SEM) (JSM‐6400; JEOL, Tokyo, Japan) at a magnification of 1000×. The vertical marginal discrepancy was defined as the perpendicular distance from the crown margin to the cavosurface angle of the implant abutment platform, following the definition proposed by Holmes et al. (1989).

Each specimen was positioned in a custom‐fabricated jig designed to maintain consistent orientation throughout SEM imaging. The jig ensured that the crown–abutment interface was aligned perpendicular (0° tilt) to the SEM optical axis. Positioning was standardized using the goniometric stage of the SEM and verified with a digital protractor prior to image acquisition to minimize angular variation and ensure reproducibility of vertical marginal measurements.

The buccal and lingual surfaces were permanently marked using a fine‐tip indelible marker (Lumocolor Permanent; Staedtler Mars, Nuremberg, Germany) based on standardized sample orientation during implant placement. A single high‐resolution SEM image was acquired per surface (buccal and lingual), resulting in two images per specimen. Each image was analyzed using INCA Suite 4.04 (Oxford Instruments), the SEM's built‐in calibrated measurement software, to establish a primary vertical measurement axis.

**Figure 3:**
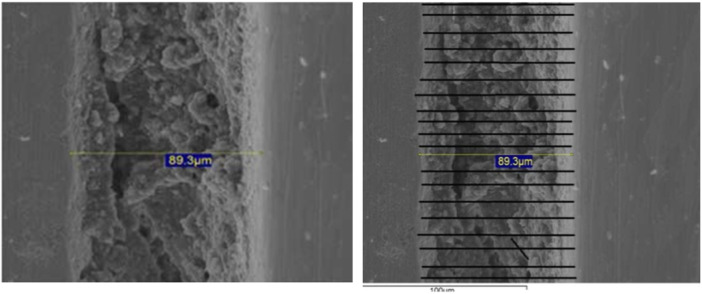
Representative SEM images of a metal‐ceramic restoration in the external connection group. (Left image) Initial vertical marginal discrepancy measurement using the SEM software's caliper tool at 1000× magnification; (Right image) digitally enhanced image with 30 equidistant parallel lines for extended measurement analysis in Adobe Photoshop.

To increase data points and spatial coverage across the margin, a digital overlay grid was applied using Adobe Photoshop (Version 2023; Adobe Inc., San Jose, CA) to generate 30 equidistant vertical lines across each image (Figure 3). The spacing of these lines was standardized at 20 pixels, based on image resolution and magnification, and maintained across all specimens. This spacing corresponded to approximately 10 µm in real scale, as determined from the SEM's embedded scale bar and verified with a certified reference grid (Ted Pella, USA). This approach provided consistent coverage of the ACI, allowing for reproducible data acquisition across all specimens and groups.

All vertical measurements were performed by a single, calibrated examiner, who was blinded to the group allocations, using a standardized protocol. Calibration was conducted prior to the study by evaluating five random samples, each sampled three times, separated by 48 h. The intra‐examiner reliability was assessed by the intraclass correlation coefficient, which exceeded 0.95, confirming high repeatability.

### 2.8 Statistical Analysis

Data were analyzed statistically using SPSS software version 22.0 (IBM SPSS Statistics, Chicago, IL). Means and standard deviations were calculated for each group. For each specimen, 30 vertical marginal measurements were taken on the buccal surface and 30 on the lingual surface, resulting in a total of 60 data points per crown. These values were then averaged per surface to obtain a mean buccal and mean lingual misfit per specimen. For statistical comparisons, each crown was treated as a single experimental unit (*n* = 10 per group), with analyses based on the averaged buccal and lingual misfit values. The Shapiro–Wilk test was used to assess the normality of the data. Since the data were normally distributed, parametric tests were applied. Differences between groups were examined using a repeated‐measures ANOVA, followed by Bonferroni post hoc tests for pairwise comparisons. Student's paired *t*‐tests were utilized to assess differences between connection types and between buccal and lingual surfaces. The significance level was set at *α* = 0.05.

## 3 Results

The VZ group exhibited the lowest marginal misfit in both EC and IC. Although the IC group showed numerically lower marginal misfit values than the EC group in some subgroups (e.g., MC and VZ), these differences were not statistically significant (*p* = 0.980), indicating no overall influence of implant connection type. Table 2 presents the vertical ACI misfit values obtained in this study.

**Table 2:** ACI misfit (μm) by material type and connection.

Group	Surface	Number	EC		IC	
			Mean	SD	Mean	SD
**MC**	B	30	49.93	16.05	47.73	22.32
	L	30	42.68	17.26	43.14	16.97
	B + L	60	46.30	16.65	45.43	19.64
**MZ**	B	30	44.83	17.66	54.53	14.91
	L	30	47.28	23.21	46.08	15.73
	B + L	60	46.05	20.43	50.30	15.11
**VZ**	B	30	26.69	14.57	23.56	14.67
	L	30	25.49	13.27	22.83	14.89
	B + L	60	26.09	13.92	23.19	14.78

Abbreviations: ACI, abutment–crown interface; B, buccal; EC, external connection; IC, internal connection; L, lingual; MC, metal‐ceramic; MZ, monolithic zirconia; SD, standard deviation; VZ, veneered zirconia.

There was no statistically significant interaction among the study variables (*p* = 0.491). Additionally, no interactions were observed between any two variables (surface‐material *p* = 0.715; surface‐connection, *p* = 0.602; material‐connection, *p* = 0.903). Therefore, each variable was analyzed separately using repeated‐measures ANOVA and paired *t*‐tests.

**Figure 4:**
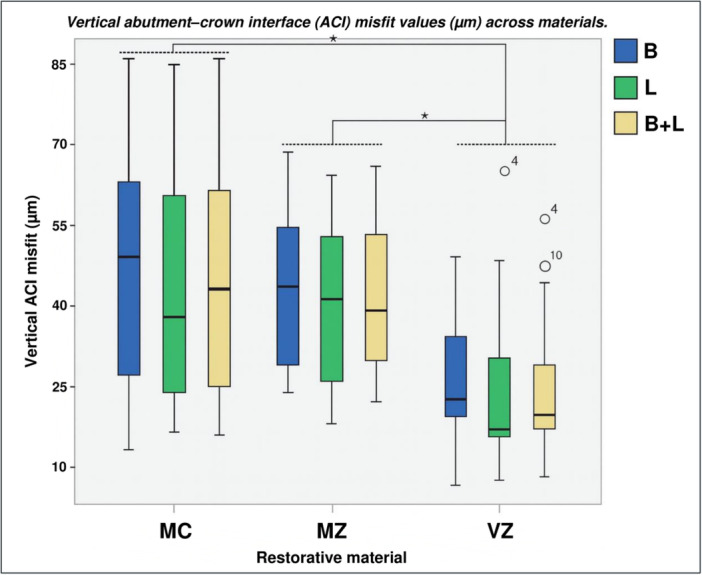
Box plot of vertical ACI misfit (μm) by restorative material (MC, MZ, VZ), shown across buccal (B), lingual (L), and combined (B + L) surfaces. Asterisks indicate statistically significant differences (*p* < 0.05, Bonferroni post‐hoc) between veneered zirconia (VZ) and both monolithic zirconia (MZ) and metal‐ceramic (MC).

The VZ group exhibited lower marginal discrepancy values on both B and L surfaces compared to the other groups (Figure 4). The one‐way ANOVA revealed differences among the analyzed materials (*p* = 0.009). The Bonferroni multiple comparison tests identified differences between the MZ–VZ (*d* = 23.54 μm, *p* = 0.016) and MC–VZ (*d* = 21.23 μm, *p* = 0.035) groups, indicating that the VZ group differed from the other material groups. Regarding discrepancies between B and L surfaces within each group, the paired *t*‐test revealed a significant difference for the MC group (*d* = 5.92 μm, *p* = 0.025).

**Figure 5:**
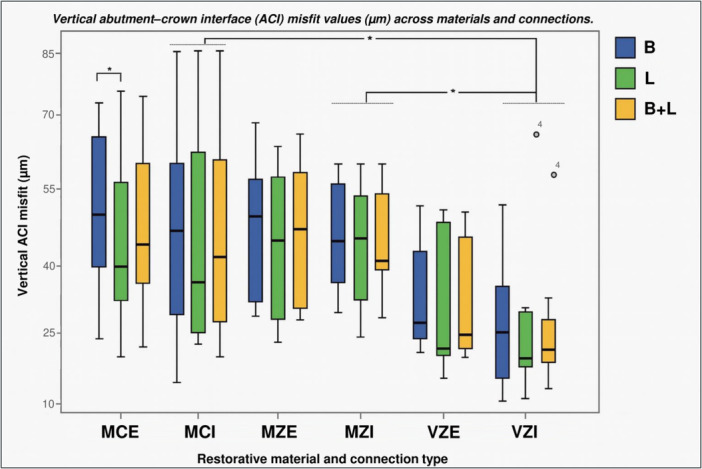
Box plot of vertical ACI misfit (μm) grouped by restorative material and implant connection type (IC, EC), shown across buccal (B), lingual (L), and combined (B + L) surfaces. Asterisks indicate statistically significant differences (*p* < 0.05) between veneered zirconia (VZ) and other materials within the internal connection group (IC). An asterisk also denotes a significant difference between buccal and lingual surfaces in the MC group within the external connection group (EC). MCE, metal‐ceramic with external connection; MCI: metal‐ceramic with internal connection; MZE, monolithic zirconia with external connection; MZI, monolithic zirconia with internal connection; VZE, veneered zirconia with external connection; VZI, veneered zirconia with internal connection.

By connection type, differences were only observed for IC (*p* = 0.02). The Bonferroni multiple comparisons test identified differences between the MZ–VZ (*d* = 27.11 μm, *p* = 0.016) and MC–VZ (*d* = 22.24 μm, *p* = 0.035) groups. When evaluating marginal misfit by connection type, irrespective of material, the IC group exhibited the smallest discrepancy (Table 3). Figure 5 illustrates the marginal misfit in each group, categorized by material and connection type.

**Table 3:** ACI misfit (μm) by connection.

Group	Surface	Number	Mean	SD
**IC**	B	90	41.94	15.35
	L	90	37.35	12.39
	B + L	180	39.64	13.87
**EC**	B	90	40.48	18.62
	L	90	38.48	26.44
	B + L	180	39.48	22.53

Abbreviations: ACI, abutment–crown interface; B, buccal; EC, external connection; IC, internal connection; L, lingual; SD, standard deviation.

Paired *t*‐test showed no differences between IC and EC (*d* = 0.16 μm, *p* = 0.980) (Table 3). A post hoc power analysis was performed for this comparison, yielding a statistical power of 5.1% based on the observed effect size (Cohen's *d* = 0.009). Similarly, no differences were found between B and L surfaces (*d* = 3.3 μm, *p* = 0.188). However, within each connection type, a difference was observed for EC (*d* = 2 μm, *p* = 0.005) (Table 3). Additionally, within the MC group, differences were found for EC (*d* = 7.25 μm, *p* = 0.007).

## 4 Discussion

This study aimed to evaluate the marginal fit at the ACI across different restorative materials and implant connection types. While the primary interface of interest was the ACI, implant connection configuration (internal vs. external) was included to investigate whether it could indirectly influence crown fit through its effect on abutment stability and positioning. Although the crown is seated on a prefabricated abutment, the implant–abutment connection determines the vertical stop, rotational resistance, and mechanical stability of the abutment under torque. In particular, internal connections may provide a more reproducible axial seating due to their deeper engagement and frictional locking. This could potentially reduce minor vertical discrepancies during abutment placement, which may subsequently influence crown seating and marginal adaptation during cementation. Even in the absence of mechanical loading, these geometric differences could subtly affect marginal fit (Diaz et al. 2025; Pjetursson et al. 2018; Vinhas et al. 2020). The results led to a partial rejection of the null hypothesis, which posited that there would be no significant differences in marginal misfit among restorative materials or between implant connection types. While the implant connection type showed no statistically significant effect on marginal fit, veneered zirconia restorations exhibited significantly lower marginal discrepancies compared to monolithic zirconia and metal‐ceramic restorations.

One of the major challenges in marginal fit analysis is the lack of standardization in both in vitro and in vivo studies. Variability in sample sizes, finish lines, timing of measurements (before or after cementation), cementation techniques, storage conditions, and measurement methods contributes to inconsistent findings across studies (Ayres et al. 2024; Diaz et al. 2025; Gonzalo et al. 2009a, 2009b; Gonzalo et al. 2020; Hasanzade et al. 2021; Katsoulis et al. 2017; Mai et al. 2020; Pan et al. 2021). Various techniques have been used to assess ACI misfit, including direct vision (DV) methods such as SEM, stereomicroscopy, optical microscopy, and scanning laser microscopy, as well as cross‐sectional methods and computed microtomography (CTM) for in vitro studies. In clinical settings, techniques such as the triple scan method (TSM), dual scan method, silicone replica technique (SRT), and optical coherence tomography (OCT) have been employed (Ayres et al. 2024; Pan et al. 2021). DV using SEM remains the most commonly used method, and previous studies suggest that SEM provides one of the most accurate representations of marginal misfit, particularly when compared with indirect replica techniques (Ayres et al. 2024; Gonzalo et al. 2009a; Ispas et al. 2023; Ortega et al. 2017; Pan et al. 2021). To ensure measurement consistency, specimens were securely positioned, and all images were captured at a fixed magnification and angulation (30°–35°) to align the interface perpendicular to the microscope's optical axis. However, SEM accuracy may still be influenced by lens surface angles, focus ranges, working distances, and magnification settings, leading to potential measurement errors of up to 10% (Gonzalo et al. 2009b; Lopez‐Suarez et al. 2016; Ortega et al. 2017). Recent evidence suggests that digital techniques such as CTM, OCT, and optical scanning may offer higher accuracy by creating a digital representation of the prosthesis‐abutment interface (Liang et al. 2023; Mai et al. 2020; Pan et al. 2021). Measurement points in this study were selected based on Holmes et al.'s (1989) method, which considers contour differences between the abutment margin and the cavosurface angle of the crown. Research has shown that using multiple measurement points is essential to avoid bias due to local morphological variations (Park et al. 2016). Groten et al. (2000) recommended a minimum number of 50 measurements, while Daou (2022) suggested performing 60 measurements for a comprehensive representation of ACI misfit. Accordingly, this study conducted 60 measurements per restoration (30 on buccal and 30 on lingual surfaces), ensuring consistency in measurement.

There is no universal consensus on an acceptable clinical threshold for ACI misfit. McLean and Von Fraunhofer proposed a 120 μm threshold for successful restorations (McLean and von 1971). Witkowski et al. (2006) reported that ceramic crowns exhibit marginal gaps ranging from 123 to 154 μm, while other studies suggest that values of ≤ 100 μm is clinically optima (Lopez‐Suarez et al. 2016; Ortega et al. 2017). Zarone et al. (2019) consider a marginal fit acceptable ≤ 75 μm. The findings of this study found marginal discrepancies below 50 μm for all tested materials, indicating clinically acceptable adaptation.

Clinical studies indicate that MZ exhibits superior marginal fit compared to MC (Paul et al. 2020) and lithium disilicate crowns (Kakroo et al. 2024; Nawafleh et al. 2023). Additionally, VZ crowns demonstrate greater ACI misfit than MZ crowns (Jalalian and Rastin 2022). Temizkan Nizaroglu and Küçük (2024) reported mean marginal gaps of 60.24 μm and 68.23 μm for two types of MZ crowns, compared to 83.66 μm for VZ crowns. Fasih et al. (2023) identified significant differences in marginal adaptation using the SRT and TSM techniques, which were further corroborated by subsequent studies (Amuthavalli et al. 2020; Mohaghegh et al. 2020). The veneering technique significantly influences marginal misfit, with increases of up to 25% depending on the method used (Temizkan Nizaroglu and Küçük 2024). Press‐over and CAD‐on techniques result in smaller ACI misfit increases than conventional layering (Abduo et al. 2023; Elter et al. 2024). Other contributing factors include impression accuracy, porcelain shrinkage, thermal mismatches due to differences in the CTE, and repeated heating and cooling cycles (Abduo et al. 2023; Alsarani et al. 2023; Torabi et al. 2015). Porcelain shrinkage exerts compressive forces on the coping, while CTE mismatches induce tensile stress during cooling, both of which influence marginal adaptation (Al‐Baadani 2016; Miura et al. 2014). In this study, the VZ group exhibited the lowest ACI misfit, likely due to the inherent strength of zirconia copings, which may resist distortion from porcelain firing shrinkage (Saraswathi et al. 2016). Unlike MZ, which undergoes a single sintering cycle with significant volumetric shrinkage, the VZ framework benefits from multiple controlled firing cycles, allowing for minor dimensional adjustments and reducing marginal discrepancies (Yang et al. 2023). Additionally, the veneering process applies compressive forces to the zirconia substructure, thereby enhancing its adaptation (Abduo et al. 2023). Conversely, MC restorations with hard‐milled metal copings may exhibit greater misfit due to residual internal stresses from milling and the CTE mismatch (Usta Kutlu and Hayran 2024; Yang and Li 2024). The optimized processing of VZ likely contributed to its superior marginal adaptation. The lack of significant influence of implant connection type on ACI misfit can be attributed to the use of precisely machined, prefabricated titanium abutments and standardized CAD‐CAM fabrication protocols, which provide high precision in marginal adaptation.

Although cement influences vertical ACI misfit (Kale et al. 2016; Martinez‐Rus et al. 2013), self‐adhesive resin cement was used to closely simulate clinical conditions. It is now well established that the most critical parameter affecting this process is the cement space. Studies by Kale et al. (2016) and Alkhallagi et al. (2023) reported that increasing the cement space up to 70 μm did not compromise the vertical marginal adaptation of MZ crowns. The increased space reduces hydraulic pressure, allowing for improved material flow and enhanced prosthesis seating. Based on previous research, a relief of 50 µm was selected for all cases (Alkhallagi et al. 2023; Gonzalo et al. 2009b; Kale et al. 2016; Lopez‐Suarez et al. 2016; Ortega et al. 2017). To date, no studies have examined differences in the vertical ACI misfit of implant connections.

It is important to distinguish between statistical significance and clinical superiority. Although the differences in marginal misfit between groups reached statistical significance, all values remained well below the most widely accepted clinical thresholds for marginal adaptation. This suggests that, from a clinical perspective, all tested restorative materials provided acceptable adaptation at the ACI. Therefore, while VZ exhibited the lowest marginal misfit, the clinical relevance of these differences should be interpreted with caution. In clinical practice, material selection may also consider additional factors, such as aesthetic requirements, fracture resistance, and veneering techniques, especially in high‐load or aesthetically demanding zones.

This in vitro study has several methodological limitations that should be acknowledged when interpreting the results. First, although SEM imaging provides high‐resolution visualization of the ACI, the measurement process involves manual analysis using image overlay software, which is inherently user‐dependent. While intra‐examiner repeatability was established and calibration protocols were followed, this technique remains susceptible to subjective error in line placement and edge detection. Future studies could benefit from the use of automated metrology or edge‐detection software to enhance measurement objectivity and reproducibility. Second, marginal misfit was assessed only at the buccal and lingual surfaces. This two‐point approach was chosen to standardize specimen positioning and SEM image acquisition, but it does not provide a complete circumferential assessment of the crown margin. Misfits at mesial and distal surfaces may differ due to anatomical or processing variability and should be evaluated in future studies using four‐surface or full 3D scanning techniques. Third, although thermal cycling was performed to simulate intraoral aging, the specimens were not subjected to mechanical loading or dynamic fatigue. Functional forces, such as masticatory load, could influence marginal stability and long‐term adaptation of the crown–abutment interface, particularly when different materials and connection geometries are involved. While the findings contribute to the understanding of how restorative materials and connection designs affect marginal adaptation under controlled conditions, their applicability to clinical scenarios remains limited due to the absence of biological and functional variables.

## 5 Conclusion

Within the limitations of this study, the results indicate that the type of implant connection did not influence the marginal misfit. Veneered zirconia restorations exhibited the lowest marginal misfit at the crown‐abutment interface in both internal and external connections, with values below 25 µm in the internal connection, indicating high adaptation precision.

## Author Contributions

Conceptualization: Pedro Diaz, Barbara Miegimolle, Antonio Martin, Celia Tobar, and Maria J. Suarez. Methodology: Pedro Diaz, Barbara Miegimolle, Antonio Martin, Celia Tobar, and Maria J. Suarez. Software: S. Antonio Martin. Validation: Maria J. Suarez. Formal analysis: Pedro Diaz, Barbara Miegimolle, and Antonio Martin. Investigation: Pedro Diaz and Antonio Martin. Resources: Maria J. Suarez and Pedro Diaz. Data curation: Pedro Diaz. Writing—original draft preparation: Pedro Diaz, Barbara Miegimolle, Celia Tobar, and Maria J. Suarez. Writing—review and editing: Pedro Diaz, Barbara Miegimolle, Antonio Martin, S. Antonio Martin, Celia Tobar, and Maria J. Suarez. Visualization: S. Antonio Martin. Supervision: Maria J. Suarez. Project administration: Maria J. Suarez. Funding acquisition: Maria J. Suarez. All authors have read and approved the published version of the manuscript.

## Ethics Statement

The authors have nothing to report.

## Consent

The authors have nothing to report.

## Conflicts of Interest

The authors declare no conflicts of interest.

## Data Availability

The data presented in this study are available upon request from the corresponding author.
